# Epigenetics of Cutaneous T-Cell Lymphomas

**DOI:** 10.3390/ijms23073538

**Published:** 2022-03-24

**Authors:** Natsumi Hara, Yu Sawada

**Affiliations:** Department of Dermatology, University of Occupational and Environmental Health, Kitakyushu 807-8555, Japan; hrntsmkf@med.uoeh-u.ac.jp

**Keywords:** epigenetic change, cutaneous T-cell lymphoma, histone

## Abstract

Epigenetic modifications rarely occur in isolation (as single “epigenetic modifications”). They usually appear together and form a network to control the epigenetic system. Cutaneous malignancies are usually affected by epigenetic changes. However, there is limited knowledge regarding the epigenetic changes associated with cutaneous lymphomas. In this review, we focused on cutaneous T-cell lymphomas such as mycosis fungoides, Sézary syndrome, and anaplastic large cell lymphoma. With regard to epigenetic changes, we summarize the detailed chemical modifications categorized into DNA methylation and histone acetylation and methylation. We also summarize the epigenetic modifications and characteristics of the drug for cutaneous T-cell lymphoma (CTCL). Furthermore, we discuss current research on epigenetic-targeted therapy against cutaneous T-cell lymphomas. Although the current method of treatment with histone deacetylase inhibitors does not exhibit sufficient therapeutic benefits in all cases of CTCL, epigenetic-targeted combination therapy might overcome this limitation for patients with CTCL.

## 1. Introduction

Epigenetic changes are well-known regulatory mechanisms related to external environmental factors [1]. These changes, such as methylation and acetylation, are involved in the chemical modification of DNA and DNA-binding proteins, particularly histones. In particular, DNA hypermethylation is often observed in lymphoma. These changes alter chromatin structure and manipulate targeted gene expression to avoid changing DNA sequence information [1]. Various environmental factors can induce chemical modifications via environmental chemicals and microorganisms [2]. Therefore, it is essential to obtain current and updated knowledge on epigenetics to better understand the development of cutaneous lymphomas. Representative epigenetic modifications observed in CTCL are as follows [1]:

### 1.1. DNA Methylation

DNA methylation is an epigenetic process of methyl groups modification to the DNA molecules [3]. Methylation changes the DNA segment activity without altering the DNA sequence information [4]. DNA methylation takes place in two nucleobases, adenine and cytosine [5]. DNA methylation is mainly observed in CpG dinucleotides, which are enriched in with the cytosines on both DNA strands [6]. DNA methylation targets the CqG island, which is often observed in gene promoter sites, as this site enriches DNA regions with a cytosine nucleotide followed by a guanine nucleotide in a linear sequence from the 5′ to 3′ direction [6,7]. As a result of DNA methylation, the targeted gene expression was silenced.

### 1.2. Histone Methylation

Histone methylation is a biological process showing methyl groups transferred to amino acids of histone proteins [8,9]. Histone methylation enhances the weakened chemical connection between DNA and histone tails [10]. In that case, histone methylation positively drives gene transcription because the uncoiled DNA from nucleosomes can access transcription factor proteins and RNA polymerase [10]. Histone methylation influences histone H3 lysine residues and initiates the activation and repression of gene transcription [11]. Histone methyltransferase enhances histone methylation. In contrast, histone demethylases cancel histone methylation.

### 1.3. Histone Acetylation

Histone acetylation is a process of the acetylation of lysine residues within the N-terminal tail from the histone core [12]. Acetylation impairs the positive charge on the histones and decreases the voltage interaction of histones with the negatively charged DNA [12], and this alteration causes a more relaxed chromatin structure, which enhances gene transcription [12]. Histone acetylation influences the lysine residues of histones for the cancellation of voltage-charge connections between DNA and histones, leading to the activation of gene transcription [1]. Histone acetyltransferase accelerates histone acetylation, whereas histone deacetylase (HDAC) suppresses histone acetylation to repress gene transcription [13].

### 1.4. MicroRNA (miRNA)

miRNAs are recognized as small non-coding RNAs exhibiting an average of 22 nucleotides in length [14]. Most miRNAs are transcribed from DNA sequences into primary miRNAs and processed into precursor miRNAs and mature miRNAs [14]. Most miRNAs interact with the 3′ UTR of target mRNAs to suppress expression [15]. miRNAs are epigenetic modulators that influence the protein levels of target miRNAs without modifying the gene sequences [16]. Furthermore, miRNAs can indirectly influence epigenetic modifications such as DNA methylation and histone modifications [16].

## 2. Cutaneous T-Cell Lymphomas

Mycosis fungoides is the most common malignant lymphoma of the skin, accounting for half of the primary cutaneous lymphomas [17]. Tumor cells show intraepidermal infiltration, which usually progresses gradually from the erythematous to the flat infiltration stage and finally to the tumor stage over several years or decades [18,19]. Early stage mycosis fungoides show favorable clinical behavior, and the 5-year survival rate of stage IA is close to 90% [20,21]. The tumor stage of mycosis fungoides is associated with a particularly poor prognosis, as metastasis to the lymph nodes is common [21]. Topical steroids and ultraviolet light therapy have been primarily selected as initial treatments [21]. Multi-drug chemotherapy is the main course of treatment for patients with an advanced form of mycosis fungoides, and epigenetic-targeted treatment is currently being explored as a therapeutic option against mycosis fungoides, such as the HDAC inhibitor vorinostat alone, in clinical trials [22].

Erythroderma and tumor cell infiltration into peripheral blood (Sézary cells) are clinical features of Sézary syndrome [21]. The pathological findings of this disease are similar to those of mycosis fungoides; however, epidermotropism seems to be mild compared to mycosis fungoides. The prognosis of Sézary syndrome is unfavorable, showing approximately a 20% 5-year survival rate [20]. Currently, there is no effective treatment for this disease; however, bone marrow cell transplantation can be conducted in a limited number of cases because of its high incidence in the elderly population [23].

Primary cutaneous anaplastic large cell lymphoma is classified as a primary cutaneous CD30-positive lymphoproliferative disease characterized by solid nodules and tumors in the skin and is mostly observed in the elderly population [24]. The tumor cells densely infiltrate the skin without epidermotropism, and over 75% of the cells show CD30 immunoreactivity. The prognosis is relatively favorable, with a 5-year survival rate of 76–96% [25].

## 3. Epigenetics Modification in CTCL

### 3.1. DNA Hypermethylation in Cyclin Dependent Kinase Inhibitor 2A (CDKN2A)

p16 is encoded by *CDKN2A*, which is recognized as a tumor suppressor gene and is frequently deleted and mutated in various malignancies. Furthermore, it plays an important role in tumor development. p16 is often inactivated in the genomes of patients with malignancies [26], and methylation of the DNA promoter site is one of its silencing mechanisms. p16 is involved in the inactivation of the cyclin D-cyclin-dependent kinase 4 complex, leading to the suppression of retinoblastoma protein and regulation of cell-cycle protein transcription and cell proliferation. A genome-wide DNA methylation screening analysis identified DNA hypermethylation in the promoter region of *CDKN2A* in 33% of the patients with CTCL [27]. In fact, p16 inactivation has been found in patients with mycosis fungoides [28] and is frequently observed during the tumor stage (77.8%) compared to the plaque stage of mycosis fungoides (44.4%) [29]. This finding suggests that p16 is responsible for tumor suppression in patients with mycosis fungoides. Furthermore, 5-aza-2′-deoxycytidine (5-aza-CdR) is a DNA methyltransferase inhibitor that reactivates silenced genes [30] and exhibits antitumor activity against anaplastic large cell lymphoma (ALCL) by inducing apoptosis and cell cycle arrest mediated by demethylation and activation of p16 following drug treatment [31].

### 3.2. DNA Hypermethylation in PYD and CARD Domain Containing (PYCARD)

*PYCARD* encodes the protein TMS1, which was identified as a protein that forms aggregates in human leukemia cells during treatment with chemotherapeutic agents, such as etoposide and cisplatin [32]. The TMS1 protein is encoded by *PYCARD*, which is silenced by DNA methylation mechanisms in various malignancies, leading to the prevention of tumor cell apoptosis, which is why it is known as a tumor suppressor gene [33].

DNA methylation of the *PYCARD* promoter site has been reported in 10% of patients with CTCL [27]. TMS1 may have antitumor effects against CTC; however, the detailed function of TMS1 in CTCL development remains unclear.

### 3.3. DNA Hypermethylation in Peroxisome Proliferator Activated Receptor Gamma (PPARG)

**Figure 1:**
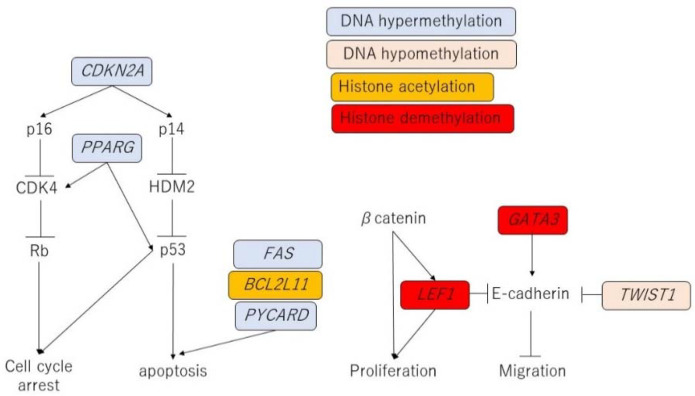
Signal transduction and epigenetic modification network in CTCL. DNA hyper- and hypomethylation and histone acetylation and deacetylation influence the expression of targeted genes and development of tumor via signal transduction. *CDKN2A*: Cyclin Dependent Kinase Inhibitor 2A; *PPARG*: Peroxisome Proliferator Activated Receptor Gamma; *CKD4*: Cyclin Dependent Kinase 4; *HDM2*: Human Double Minute2; *Rb*: Retinoblastoma; *FAS*: Fas cell Surface Death Receptor; *BCL2L11*: BCL2 Like 11; *PYCARD*: PYD and CARD Domain Containing; *LEF1*: Lymphoid Enhancer-Binding Factor 1; *GATA3*: GATA Binding Protein 3; *TWIST1*: Twist Family bHLH Transcription Factor 1.

PPAR is a member of the nuclear receptor superfamily and is activated in a ligand-dependent manner to regulate the expression of target genes (Figure 1). PPAR has three subtypes: PPAR-α, PPAR-δ, and PPAR-γ. PPAR-γ ligands suppress the production of inflammatory cytokines [34,35] and growth of cultured CRC cells [36]. Therefore, *PPARG* is expected to positively drive antitumor activity. The actual role of *PPARG* in cutaneous lymphoma remains unclear; however, *PPARG* hypermethylation is a significant predictor of disease progression [37]. The downregulation of *PPARG* by hypermethylation may positively contribute to the development of CTCL [37].

### 3.4. DNA Hypomethylation in Fas Cell Surface Death Receptor (FAS)

Mycosis fungoides and Sézary syndrome are characterized by the inhibition of cell apoptosis. Upregulation of the Fas ligand plays a central role in tumor cell apoptosis, mediated by subsequent apoptosis through the Fas death receptor pathway. Although CTCL cells repress the expression of *FAS*, it could be epigenetically increased via the de-repression of the *FAS* promoter by DNA hypomethylation, which can enhance tumor cell apoptosis in patients with CTCL [38]. Sézary syndrome shows hypermethylation in the promoter region of *FAS*, resulting in downregulation of the gene expression [39].

### 3.5. DNA Hypomethylation in Twist Family bHLH Transcription Factor 1 (TWIST1)

*TWIST1* is a basic helix-loop-helix (bHLH) transcription factor that is essential during embryonic development and is associated with tumor metastasis and growth [40,41]. It also promotes cancer stem cell development and tumorigenesis. DNA hypomethylation at the promoter site of *TWIST1* in patients with Sézary syndrome increases [42], which is closely related to unfavorable clinical behavior associated with mycosis fungoides [42,43]. Hypomethylation of the *TWIST1* promoter contributes to tumorigenesis and tumor development [42,43].

### 3.6. Histone Acetylation in BCL2 like 11 (BCL2L11)

BIM is a pro-apoptotic member of the Bcl-2 family and is recognized as a regulatory protein for apoptosis. BIM plays a critical role in the suppression of oncogenesis as a tumor suppressor and impairs tumor metastasis and apoptosis. In patients with ALCL, the BIM is encoded by *BCL2L11*, which is suppressed due to the recruitment of the SIN3a–HDAC1/2 corepressor complex, and treatment with the deacetylase inhibitor trichostatin This reverses this suppression and enhances tumor cell apoptosis [44].

### 3.7. The Enhancer of Zeste Homolog 2 (EZH2)-Mediated Histone Methylation

EZH2 is a component of polycomb repressive complex 2 and mediates histone H3 lysine 27 trimethylation. Highly upregulated EZH2 is recognized in patients with ALCL and large-cell-transformed cutaneous T-cell lymphoma [45]. EZH2 promotes disease progression through histone methyltransferase activity, which represses cell apoptosis and facilitates cell-cycle progression in primary cutaneous ALCL [45]. As the mechanism of the molecular mechanisms leading to EZH2 upregulation in ALCL, it is speculated that EZH2 overexpression in PCALCL may be a downstream event of MYC activation [45]. EZH2 expression in cancers is affected by multiple pathways, including N-MYC [46] and C-MYC [47]. Furthermore, MYC has been reported to play a pivotal role for ALCL survival [48], suggesting the presence of an MYC-mediated EZH2 regulation mechanism.

### 3.8. Histone Demethylation in GATA Binding Protein 3 (GATA3)

GATA3 is a transcription factor belonging to the GATA family of proteins, which plays an essential role in the differentiation of various cell types [49]. Consistently, high *GATA3* expression has been associated with unfavorable clinical behavior in patients with peripheral T-cell lymphoma [50]. Suppressive trimethylation of histone H3 lysine 27 of *GATA3* is recognized in tumors of patients with ALCL [51]. Histone demethylation of *GATA3* promotes gene expression and may contribute to the development of ALCL. Therefore, histone demethylase inhibitors may be advantageous in mediating histone demethylation of *GATA3* during the treatment of patients with CTCL.

### 3.9. Histone Demethylation in Lymphoid Enhancer-Binding Factor 1 (LEF1)

*LEF1* is a member of the T-cell factor (TCF)/LEF1 family of high-mobility group transcription factors and is a mediator of the Wnt/β-catenin signaling pathway. It is essential for the maintenance of stem cell and organ development and has been identified in patients with mycosis fungoide and Sézary syndrome [52]. Histone demethylation enhances *LEF1* expression in ALCL cells [51]. This epigenetic alternation is expected to drive tumor proliferation by enhancing β-catenin signaling [53] by silencing *LEF1* expression.

### 3.10. miRNA in CTCL

miRNAs negatively regulate gene expression, and aberrant expression is involved in the development of CTCL. Tumor-stage mycosis fungoides show upregulated levels of onco-miRNAs, such as miR-146a, miR-142-3p/5p, miR-21, miR-181a/b, and miR-155; and downregulated levels of tumor-suppressor miRNAs, such as miR-200ab/429 cluster, miR-10b, miR-193b, miR-141/200c, and miR-23b/27b [54]. Patients with ALCL show overexpression of miR-155, miR-21, or miR-142-3p/5p, and underexpression of miR-141/200c [54]. miRNAs are also involved in the pathogenesis of CTCL, mediated by the regulation of Notch1 signaling. Although there is no detection of DNA methylation at the Notch1 promoter, methylation of miR-200c has been observed in cases of mycosis fungoides [55]. The downregulation of miR-200c mediated by methylation is associated with the overexpression of Jagged1, which is responsible for Notch1 activation, hence the development of mycosis fungoides [55].

### 3.11. Possible Linkage between Epigenetic Modifications

To better understand the influence of epigenetic alterations, we summarized the signal pathway interactions for each epigenetic modification target (Figure 1). *CDKN2A* mediates p16/CDK4/Rb and p14/HDM2/p53 signal transduction [56]. *PPARG* is also involved in CDK4-mediated activation of Rb and p53 [57,58]. Therefore, hypermethylation of *CDKN2A* and *PPARG* can impair gene expression during the development of tumors. FAS, *BLC2L11*, and *PYCARD* are responsible for the initiation of tumor cell apoptosis. These epigenetic modifications impair the induction of apoptosis in tumor cells. β-catenin plays an essential role in tumor cell proliferation, and *LEF1* cooperates with β-catenin to accelerate tumor development [53]. *GATA3* and *TWIST1* are involved in the E-cadherin expression [53,59,60], which determines how potent the tumor cell migration is. Targeted gene alterations by epigenetic modifications negatively regulate E-cadherin expression and enhance tumor cell migration.

### 3.12. The Therapeutic Target for Epigenetics in Cutaneous Lymphomas

The epigenetic alteration of gene transcription using HDAC inhibitors is a novel strategy for cancer therapy. Vorinostat is an HDAC inhibitor approved by the FDA for the treatment of patients with CTCL. It shows an overall response rate of 24–30% in patients with refractory advanced CTCL [61]. This drug is currently used for the treatment of cutaneous lymphoma and shows efficacy in refractory and advanced lymphomas [62]. Romidepsin is another HDAC inhibitor, and clinical trials have been performed to determine its efficacy against CTCL. An overall response rate was observed in 34% of patients, and the median duration of response was 13.7 months [63]. A clinical trial of the HDAC inhibitor belinostat was conducted in patients with relapsed or refractory peripheral or cutaneous T-cell lymphoma, and the objective response rate was 14% in patients with CTCL [64]. Histone acetylation in *BCL2L11* is responsible for the apoptosis of CTCL tumor cells. Therefore, HDAC inhibitors are expected to induce apoptosis mediated by the acceleration of histone acetylation in these targeted genes.

A DNA methyltransferase inhibitor (DNMTi, 5-Azacytidine) can reduce tumor proliferation by regulating human telomerase reverse transcriptase gene (hTERT) expression [65], which is responsible for telomere maintenance and tumorigenesis in patients with Sézary syndrome [66,67]. Therefore, DNMT inhibitors are expected to show a beneficial impact to attenuate the effects of DNA hypermethylation on *CDKN2A, PPARG, FAS,* and *PYCARD.* In contrast, DNA hypomethylation in *TWIST1* is involved in the development of CTCL, suggesting that DNA methylation-targeted treatment might have difficulty regulating both hypomethylation and hypermethylation of DNA by chemical agents and could be a reason for the limited number of studies regarding the efficacy of DNMT inhibitors against CTCL.

Recent studies have demonstrated the effects of combination therapy with an HDAC inhibitor. Vorinostat plus plicamycin treatment induced apoptosis ex vivo and showed a synergistic antitumor effect against Sézary syndrome cells [68]. The bromodomains of bromodomain and extra-terminal motif (BET) inhibitors prevent protein–protein interactions between BET proteins, acetylated histones, and transcription factors [69]. The combination of BET and HDAC inhibitors synergistically induces cell cycle arrest and apoptosis [70]. Epigenetic alterations associated with CTCL are also observed in aberrant DNA gene methylation. Therefore, the combination of a DNA demethylating agent and hydralazine with an HDAC inhibitor showed additional therapeutic effects against mycosis fungoides responding to hydralazine and valproate, two repositioned drugs, such as HDAC and DNA methylation inhibitors, respectively [71]. Because no clinical trial has evaluated the efficacy of DNMTi for the treatment of CTCL, further investigation is required.

Histone demethylase inhibition has been reported to have antitumor effects in patients with CTCL. JIB-04 is currently used to evaluate the efficacy of histone demethylase inhibitors in the treatment of malignancies [72,73]. As there are no clinical trial outcomes, further investigation is required to evaluate the therapeutic efficacy of CTCL treatment.

Considering the problems involved in epigenetics-targeted treatment, the current therapeutic options, including HDAC inhibitors, do not show therapeutic efficacy in all patients [74], indicating that it is necessary to develop an additive therapeutic strategy using HDAC inhibitors. Therefore, treatment with immune checkpoint inhibitors may be a potential therapeutic option. The immune checkpoint inhibitor nivolumab shows therapeutic efficacy against T-cell lymphoma [75]. Although there are no clinical trials for CTCL, the presence of exhausted T cells in tumor carriers is one of the problems associated with determining the efficacy of immunotherapy [76]. Combination therapy with HDAC and DNMT inhibitors alters the T cell exhaustion state to memory and effector T cell phenotypes [77], suggesting a possible therapeutic option against CTCL.

## 4. Conclusions

We presented the beneficial potency and limitations of epigenetic-targeted treatment for CTCL. There are many epigenetic targets in CTCL; however, these therapies are currently unavailable for the clinical treatment of patients with CTCL. However, we expect that they will be developed further and be ready for clinical use in the future. Additionally, there are certain epigenetic modification targeted agents that are currently available, especially HDAC inhibitors; however, a single epigenetic-targeted treatment results in limited therapeutic outcomes in current HDAC inhibitor treatment. This problem suggests that combination therapy with other epigenetic modifiers, chemotherapy, or immunotherapy may overcome the limitations of the current epigenetic-targeted treatment of patients with CTCL. These findings indicate that epigenetic changes interact in the tumor environment in a complex manner, and it is important to gain a better understanding of the epigenetic modifications associated with the tumor (Table 1).

## Figures and Tables

**Table 1:** Epigenetics targeted genes.

Epigenetic Changes	Targeted Gene
Hypermethylation	*CDKN2A* [27] *PPARG* [37] *PYCARD* [27] *FAS* [38]
Hypomethylation	*TWIST1* [42]
Histone acetylation	*BCL2L11* [44]
Histone demethylation	*GATA3* [51] *LEF1* [51]

## Data Availability

Not applicable.
